# Impact and Origin of Interface States in MOS Capacitor with Monolayer MoS_2_ and HfO_2_ High-*k* Dielectric

**DOI:** 10.1038/srep40669

**Published:** 2017-01-13

**Authors:** Pengkun Xia, Xuewei Feng, Rui Jie Ng, Shijie Wang, Dongzhi Chi, Cequn Li, Zhubing He, Xinke Liu, Kah-Wee Ang

**Affiliations:** 1 Department of Electrical and Computer Engineering, National University of Singapore, 4 Engineering Drive 3, 117583 Singapore; 2 Institute of Materials Research and Engineering, 3 Research Link, 117602 Singapore; 3 Department of Materials Science and Engineering, South University of Science and Technology of China, 1088 Xueyuan Road, Shenzhen, 518055, People Republic of China; 4 College of Materials Science and Engineering, Shenzhen Key Laboratory of Special Functional Materials, Nanshan District Key Lab for Biopolymer and Safety Evaluation, Shenzhen University, 3688 Nanhai Ave, Shenzhen, 518060, People Republic of China

## Abstract

Two-dimensional layered semiconductors such as molybdenum disulfide (MoS_2_) at the quantum limit are promising material for nanoelectronics and optoelectronics applications. Understanding the interface properties between the atomically thin MoS_2_ channel and gate dielectric is fundamentally important for enhancing the carrier transport properties. Here, we investigate the frequency dispersion mechanism in a metal-oxide-semiconductor capacitor (MOSCAP) with a monolayer MoS_2_ and an ultra-thin HfO_2_ high-*k* gate dielectric. We show that the existence of sulfur vacancies at the MoS_2_-HfO_2_ interface is responsible for the generation of interface states with a density (*D*_*it*_) reaching ~7.03 × 10^11^ cm^−2^ eV^−1^. This is evidenced by a deficit S:Mo ratio of ~1.96 using X-ray photoelectron spectroscopy (XPS) analysis, which deviates from its ideal stoichiometric value. First-principles calculations within the density-functional theory framework further confirms the presence of trap states due to sulfur deficiency, which exist within the MoS_2_ bandgap. This corroborates to a voltage-dependent frequency dispersion of ~11.5% at weak accumulation which decreases monotonically to ~9.0% at strong accumulation as the Fermi level moves away from the mid-gap trap states. Further reduction in *D*_*it*_ could be achieved by thermally diffusing *S* atoms to the MoS_2_-HfO_2_ interface to annihilate the vacancies. This work provides an insight into the interface properties for enabling the development of MoS_2_ devices with carrier transport enhancement.

Two-dimensional (2D) MoS_2_ has attracted a lot of interests for electronics applications due to its excellent electrical properties, such as high mobility^1^, near-ideal subthreshold swing^2^ and high on/off current ratio^3^,^4^,^5^,^6^. Although experimental demonstration of transistor characteristics show a channel mobility as high as ∼1,000 cm^2^V^−1^s^−1^ at room temperature^7^, theoretical study has estimated an intrinsic mobility of ∼410 cm^2^ V^−1^s^−1^ based on first-principles calculation of electron-phonon interaction^8^. Furthermore, in high-*k* dielectric environment, the room-temperature mobility of monolayer MoS_2_ can be significantly improved due to the effective screening of Coulomb scattering. The increase in mobility using high-*k* dielectric was also observed in multilayer MoS_2_ devices, though not so significant as compared to monolayer MoS_2_^2^,^5^. The high mobility achieved through the adoption of high-*k* dielectric in MoS_2_ field-effect transistor is comparable to that of thin-film silicon^9^, thus opening up a wealth of opportunities for its application in future electronics. Moreover, high-*k* dielectric can be scaled up to achieve low gate leakage without losing the electrostatic gate control. This is crucial for its implementation in advanced technology node in which the transistor geometry will be aggressively downsized.

To integrate 2D MoS_2_ into scalable manufacturing, large-scale synthesis method to grow high-quality MoS_2_ film with precise control of thickness is essential. Bottom-up methods such as chemical vapor deposition (CVD)^10^,^11^,^12^, thermal evaporation^13^, hydrothermal synthesis^14^, electrochemical lithiation processes^15^ and sulfurization of molybdenum oxides^16^ have been introduced for fabricating large-scale layered 2D MoS_2_ on insulating substrates. Recently, a magnetron sputtering method was reported to synthesize wafer-scale, high-uniformity and high-purity MoS_2_ thin film with good control of thickness using a one-step process^17^. The thickness can be well controlled by the deposition time, substrate temperature and sputtering power. Moreover, intrinsic p-type MoS_2_ can be obtained by this method, which complements the n-type conductivity for enabling integrated circuit application.

To-date, the experimentally attainable hole mobility and on/off ratio of SiO_2_-based MoS_2_ transistors fabricated using the magnetron sputtering method have been reported to be ~12.2 cm^2^ V^−1^ s^−1^ and ~10^3^, respectively^17^. However, carrier transport study of the magnetron-sputtered MoS_2_ with an integrated high-*k* gate dielectric have not been reported so far. In particular, the electrical characteristics such as capacitance-voltage (*C-V*) frequency dispersion and its dependence on interface states density^18^ (*D*_*it*_) which influence the carrier transport properties deserve a further investigation. Apart from the mobility and on/off ratio, high *D*_*it*_ could also degrade the performance of transistor in terms of response time, traps effect on current transient, subthreshold swing and low frequency noise^19^,^20^. For methods including mechanical exfoliation (ME), CVD and thermal evaporation, the atomic defects in MoS_2_ monolayer have been systematically investigated by J. Hong *et al*.^21^. The defect density can reach up to ~10^13^ cm^−2^ and the type of the atomic defects varies for different synthesis methods. For instance, sulfur vacancies are responsible for the defects in ME and CVD while antisite defects with molybdenum replacing sulfur dominate in thermal evaporation.

Here, the impact and origin of interface states at the monolayer MoS_2_ and HfO_2_ high-*k* gate dielectric interface is investigated. We find that the presence of sulfur vacancies is responsible for the generation of interface states that causes the frequency dispersion in the accumulation regime of the MoS_2_ MOSCAP, which exhibits a dependence on the applied gate voltage. The deficit S:Mo ratio is experimentally verified by X-ray photoelectron spectroscopy (XPS) analysis. We further employ the first-principles calculations within the density functional theory framework to explain the physical origin of trap states that exist within the monolayer MoS_2_ bandgap.

## Results and Discussion

**Figure 1:**
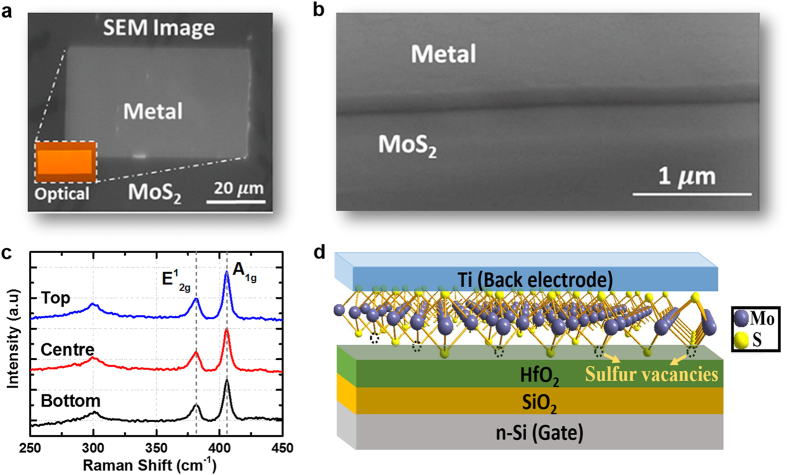
(**a**) Top view scanning electron microscopy (SEM) image of the MOS capacitor with a monolayer MoS_2_. The inset shows the optical image. (**b**) SEM image showing the metal contact edge to MoS_2_, which reveals a smooth metal/MoS_2_ interface after the lift-off process. (**c**) Raman spectra measurement of the monolayer MoS_2_ on HfO_2_ substrate. The two representative Raman characteristic bands E^1^_2g_ and A_1g_ that correspond in-plane and out-of-plane vibration modes, respectively, are found to peak at a frequency of 381.2 cm^−1^ and 405.8 cm^−1^, thus confirming the formation of MoS_2_ layer. (**d**) Schematic diagram showing the device structure of the MoS_2_ MOSCAP. A monolayer MoS_2_ nano-sheet is deposited onto the HfO_2_/Si substrate by magnetron sputtering approach. Due to an incomplete growth, sulfur vacancies are observed at the MoS_2_-HfO_2_ interface, which leads to the generation of interface states.

Figure 1(a) shows the scanning electron microscopy (SEM) image of the fabricated MoS_2_ MOSCAP. An n-type degenerately-doped silicon substrate is used as the back gate electrode and a 5 nm thick HfO_2_ is subsequently deposited by atomic layer deposition (ALD) technique. The deposition rate of the HfO_2_ films is estimated at ~1 Å/cycle, utilizing the tetrakisethylmethylamino hafnium (TEMAH), and water precursors at a deposition temperature of 250 °C for a total of 40 cycles. For every cycle, the TEMAH precursor and the water precursors are pulsed at 0.015 seconds and 0.01 seconds respectively, followed by a waiting time of 10 seconds. During the deposition, nitrogen is utilised as the carrier gas, and is flowing at 20 sccm. Following that, a large-scale monolayer MoS_2_ nanosheet is deposited by magnetron sputtering approach^17^ onto the HfO_2_/Si substrate. The MoS_2_ films are grown at high temperature (>700 °C) using Mo metal target sputtered in an vaporized sulfur ambient. Sulfur is vaporized using heating tape wrapping around the sulfur container before leaking into the chamber. The base pressure of the chamber is 3 × 10^−7^ mbar. The Argon pressure is fixed at 6.0 × 10^−4^ mbar and the sputtering power is as low as 6 W. At such low power, the growth rate is extremely low so as to achieve monolayer growth. Finally, a 100 nm Ti is deposited by e-beam evaporation as the contact electrode. Figure 1(b) depicts the SEM image showing the metal contact edge to MoS_2_, which reveals a smooth metal/MoS_2_ interface after the lift-off process. The two representative Raman-active modes E^1^_2g_ and A_1g_ with peak frequency of 381.2 cm^−1^ and 405.8 cm^−1^ confirm the formation of MoS_2_ layer^22^, as plotted in Fig. 1(c). The MoS_2_ uniformity is quantitatively verified by conducting Raman spectra measurement across a typical ~1 cm^2^ HfO_2_ substrate (i.e. top, centre and bottom spots). A schematic cross-section of the MoS_2_ MOSCAP device structure is shown in Fig. 1(d). The existence of sulfur vacancies at the MoS_2_-HfO_2_ interface are vividly shown, which is responsible for the generation of interface states.

**Figure 2:**
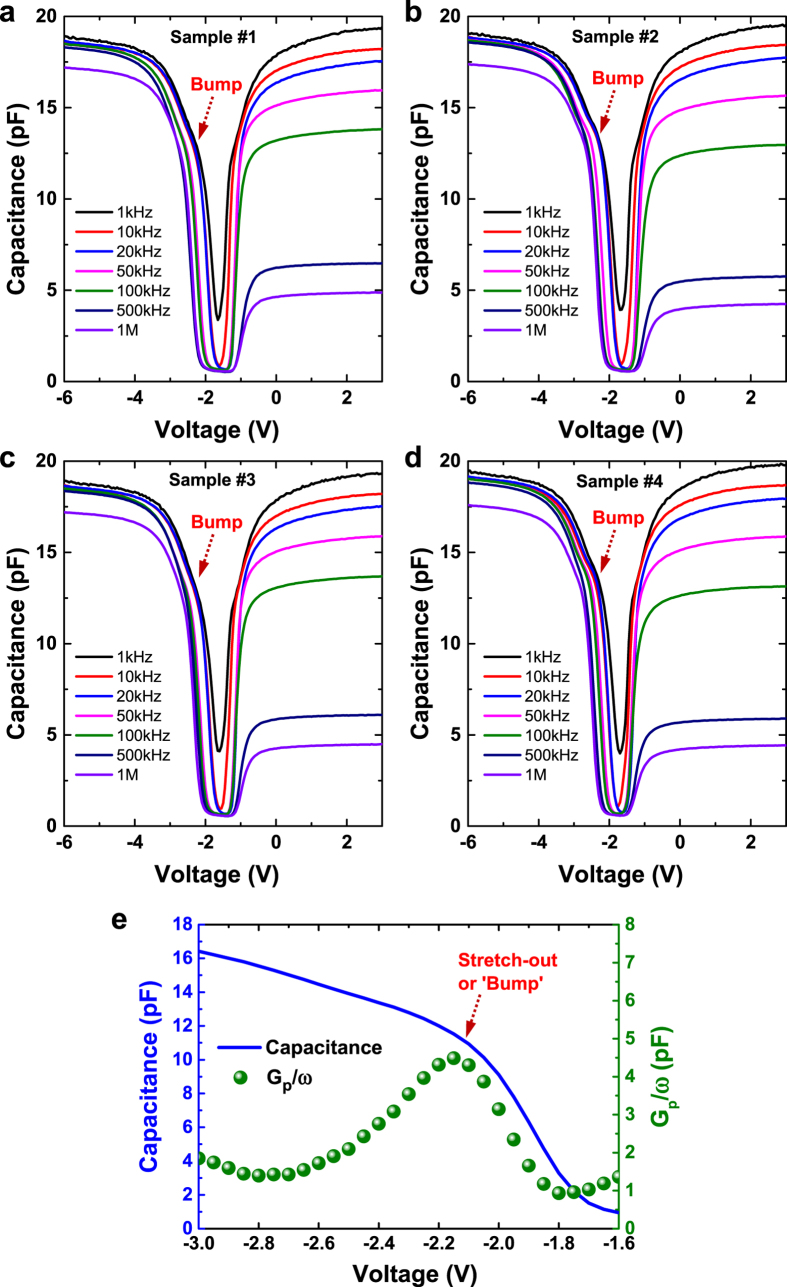
Capacitance-Voltage (C-V) characteristics of the MOSCAP with monolayer MoS_2_ measured at a range of low and high frequencies from 1 kHz to 1 MHz. (**a–d**) A repeatability check using four different samples confirms the consistency of the *C-V* measurement results. (**e**) The depletion capacitance and conductance-voltage (G_p_/ω-V) characteristics measured at a low frequency of 1 kHz. The stretch-out or bump seen in the C-V curve as a result of interface traps is evidenced by the G_p_/ω peak which unambiguously marks the activity of midgap traps that represents the losses due to the exchange of carriers with the interface traps.

**Figure 3:**
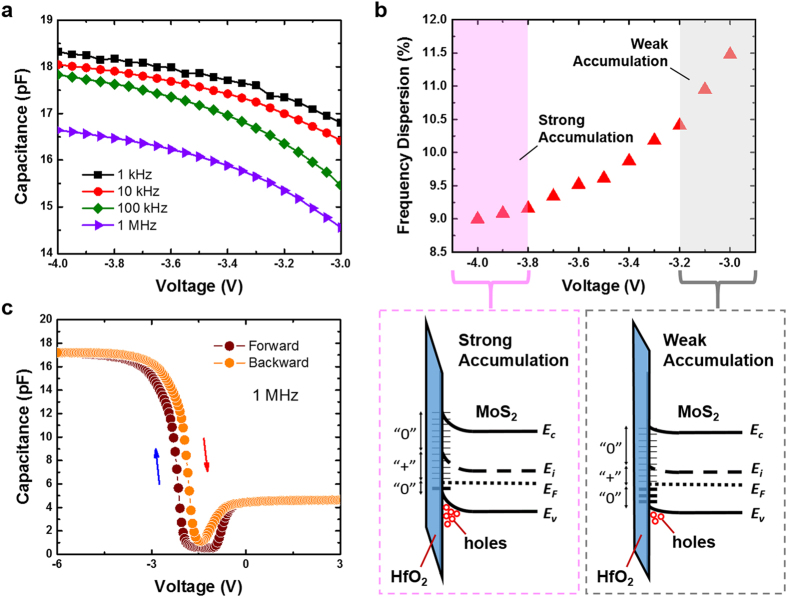
(**a**) The measured capacitance at accumulation regime decreases monotonically with increasing frequency. This is an indication of the presence of interface states (*D*_*it*_) that localized at the semiconductor/oxide interface. (**b**) The voltage-dependent frequency dispersion in the accumulation regime as defined by [*C*(1 kHz)/*C*(1 MHz)–1] × 100%. A larger average frequency dispersion of ~11.5% seen in the weak accumulation regime indicates that a more severe interface traps are present near the mid-gap of monolayer-MoS_2_ with energy levels between *E*_*F*_* < E < E*_*i*_. Hence when operates in the strong accumulation regime where the Fermi level moves away from the mid-gap traps due to an increased gate voltage, a reduction in the frequency dispersion of ~9.0% is obtained. (**c**) The small hysteresis (*ΔV*) of ~0.34 V measured in our device exemplifies the achievement of good interface between MoS_2_ on HfO_2_.

To investigate the impact of interface states on frequency dispersion, capacitance-voltage (*C-V*) characteristics of the MOSCAPs are measured using Agilent B1505A analyzer. Figure 2 plots the *C-V* curves of the monolayer MoS_2_ MOSCAPs measured as a function of frequency from 1 kHz to 1 MHz. Due to the degenerate doping in the silicon substrate (n^++^ Si) which acts as the back gate electrode, a depletion layer is typically not expected. Hence, the depletion capacitance measured in our capacitors with a Ti/MoS_2_/HfO_2_/n^++^ Si configuration is originated from the MoS_2_ layer rather than the bottom degenerately doped silicon. A repeatability check using four different samples confirms the consistency of the *C-V* measurement results [Fig. 2(a–d)]. The stretch-out or bump seen in the depletion regime is attributed to the existence of interface traps. This is further supported by the presence of G_p_/ω peak at low frequency (1 kHz), which unambiguously marks the activity of midgap traps that represents the losses due to the exchange of carriers with the interface traps. This agrees well with the correlation between conductance and interface traps, as described by Schroder D. K^23^. As can be seen in Fig. 3(a), the capacitance values are also dependent on the applied gate voltage and frequency. Notably, the measured capacitance at accumulation regime decreases monotonically with increasing frequency. This is an indication of the presence of interface states (*D*_*it*_) that localized at the semiconductor/oxide interface, which accounts for the frequency dispersion at the accumulation regime. Figure 3(b) shows the voltage-dependent frequency dispersion defined by [*C*(1 kHz)/*C*(1 MHz) –1] × 100%^24^. In weak accumulation regime, a frequency dispersion of ~11.5% is measured which decreases monotonically to ~9.0% when operates in the strong accumulation regime. To understand the mechanism, we employ energy band diagrams to describe the effect of interface traps^23^ on the frequency dispersion that exhibits a dependence on the applied gate voltage. It is worthy to note that the electron-occupied trap states are indicated by the small horizontal heavy lines and unoccupied trap states are shown by the light lines. Neutral and positively charged traps are marked by “0” and “+”, respectively. Due to electron occupancy, the donor trap states below the Fermi level (*E*_*F*_) are electrically neutral. However, those donor traps with energies above *E*_*F*_ (i.e. *E*_*F*_* < E < E*_*i*_ where *E*_*i*_ is intrinsic Fermi level) are unoccupied and hence positively charged. Conversely, trap states that reside above *E*_*i*_ are unoccupied acceptors which are electrically neutral. Therefore, the larger frequency dispersion of ~11.5% seen in the weak accumulation regime indicates that a more severe interface traps should present near the mid-gap of monolayer-MoS_2_ with energy levels between *E*_*F*_* < E < E*_*i*_. Hence when operates in the strong accumulation regime where the Fermi level moves away from the mid-gap traps due to an increased gate voltage, a reduction in the frequency dispersion of ~9.0% is achieved. In contrary, for an Al_2_O_3_/InGaAs gate stack, the dispersive behavior in accumulation has been attributed to border traps which originate from the bonding defects that exist within the gate oxide^25^. However, for the monolayer MoS_2_/HfO_2_ gate stack, given the large density of states in MoS_2_ due to a heavier hole effective mass (~2.4*m*_*o*_)^26^, the border traps capacitance would be significantly masked by the density of states capacitance. As such, the observed accumulation dispersion in our MoS_2_ MOSCAPs is unlikely to be caused by the border traps effect.

As interface trap is strongly correlated to the stretch-out of C-V curves in the depletion or weak inversion regime^27^, the *D*_*it*_ can be extracted by employing the high-low frequency (*Castagné–Vapaille*) method^28^ through the following equation


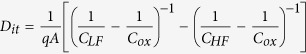


where *C*_*LF*_ and *C*_*HF*_ are the measured capacitance in the depletion or weak inversion regime at low (1 kHz) and high (1 MHz) frequency, respectively. *C*_*ox*_ is the oxide capacitance or dielectric capacitance and *q* is the elementary charge. The calculated *D*_*it*_ near the flatband voltage is determined to be ~7.03 × 10^11^ cm^−2^ eV^−1^ at the monolayer MoS_2_-HfO_2_ interface. According to previous report^29^, for multilayer MoS_2_-SiO_2_ interface with mechanical exfoliated MoS_2_ in back-gate configuration, the *D*_*it*_ can be as low as 7.2 × 10^10^ cm^−2 ^eV^−1^. However, for monolayer MoS_2_ prepared by CVD, the *D*_*it*_ at the MoS_2_-SiO_2_ interface of device with top-gate configuration can be as high as 1.6 × 10^13^ cm^−2^ eV^−1^ 30. Recently, a transfer technique used to prepare large-area, single-crystal and few-layer MoS_2_ films was reported to produce multilayer MoS_2_-SiO_2_ interface with *D*_*it*_ of 2.1 × 10^13^ cm^−2^ eV^−1^ in back-gate configuration^31^. Theoretically, due to more severe fixed charges and interface states between MoS_2_ and high-*k* dielectrics, the *D*_*it*_ should be much higher than that at MoS_2_-SiO_2_ interface. Research shows that the *D*_*it*_ at multilayer MoS_2_-Al_2_O_3_ interface can reach up to 2.6 × 10^11^ cm^−2 ^eV^−1^ and ~2 × 10^12^ cm^−2 ^eV^−1^ for back-gate^3^ and top-gate^5^,^30^ configurations, respectively, in which MoS_2_ was prepared by mechanical exfoliation^29^,^32^. Mid-gap *D*_*it*_ of ~1 × 10^12^ cm^−2 ^eV^−1^ was also reported for the CVD-grown monolayer-MoS_2_/AlO_x_/HfO_2_/Ti/Au top gate stack using capacitance and AC conductance methods^33^. However, very limited results on MoS_2_-HfO_2_ interface have been reported^34^. Here, due to a much better uniformity of the MoS_2_ film as compared to CVD grown MoS_2_^17^, we expect the quality of the dielectric and thereby *D*_*it*_ in top-gate device with MoS_2_ channel should be comparable to that of high-*k* device with MoS_2_ prepared by mechanical exfoliation. The small hysteresis (*ΔV*) of ~0.34 V measured in our device further exemplifies the good interface between the magnetron-sputtered MoS_2_ on HfO_2_, as shown in Fig. 3(c).

**Figure 4:**
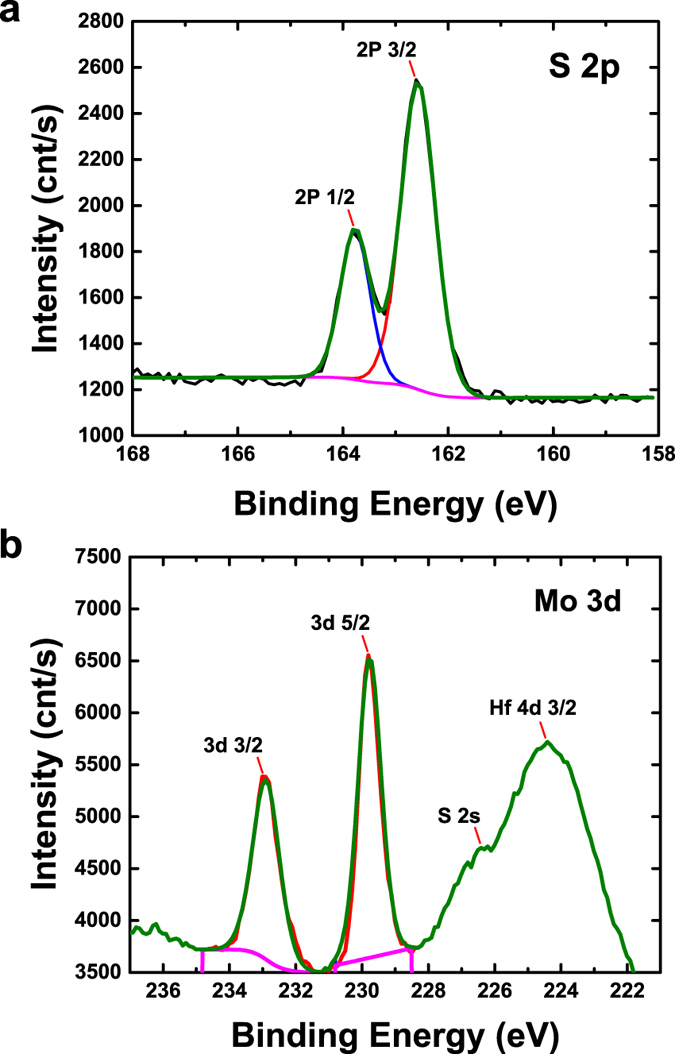
X-ray photoelectron spectroscopy (XPS) measurements are performed to analyze the MoS_2_-HfO_2_ interface properties. The deconvolution of the (**a**) S *2p* and (**b**) Mo *3d* spectra of the monolayer MoS_2_ on HfO_2_ substrate, where the binding energies of all spectra are referenced to C1s that is set to 285 eV. The doublet Mo *3d*_*5/2*_ and *3d*_*3/2*_ orbitals are found to peak at 229.77 and 232.89 eV, respectively. Whereas the spin-orbital splitting for S *2p* is well resolved into S *2p*_*3/2*_ and *2p*_*1/2*_ at 162.59 and 163.81 eV, respectively, which is in good agreement with the reported binding energy values. These XPS results confirm the formation of pure 2H-MoS_2_ crystal structure by magnetron sputtering approach. However, the extraction of S:Mo ratio shows a deficit value of ~1.96, which indicates sulfur deficiency at the interface.

To investigate the physical origin behind the generation of interface states, X-ray photoelectron spectroscopy (XPS) measurements are performed to analyze the MoS_2_-HfO_2_ interface chemistry. The core level XPS spectra are collected using a monochromatic Al Kα X-ray source with the pass energy of the analyzer set to 10 eV for high resolution measurement. The deconvolution of the S *2p* and Mo *3d* spectra of the monolayer MoS_2_ on HfO_2_ substrate are shown in Fig. 4(a,b), respectively. The binding energies of all spectra are referenced to C1s which is set to 285 eV. The doublet Mo *3d*_*5/2*_ and *3d*_*3/2*_ orbitals are found to peak at 229.77 and 232.89 eV, respectively. Whereas the spin-orbital splitting for S *2p* is well resolved into S *2p*_*3/2*_ and *2p*_*1/2*_ at 162.59 and 163.81 eV, respectively, which is in good agreement with the reported binding energy values. These XPS results confirm the formation of pure 2H-MoS_2_ crystal structure by magnetron sputtering approach. However, the extraction of S:Mo ratio shows a deficit value of ~1.96, which indicates sulfur deficiency at the interface. This is attributed to the generation of sulfur vacancies due to an incomplete growth of a monolayer MoS_2_. However, when the MoS_2_ growth proceeds to achieve multi-layer or bulk film, the high growth temperature^35^ could promote the diffusion of sulfur atoms to the MoS_2_-HfO_2_ interface to annihilate the S vacancies and reduce the interface states density. Similar method has been reported^36^ where back-gated field-effect transistors (FETs) were fabricated on two types of MoS_2_ flakes, i.e. as-exfoliated and sulfur-treated. It has been shown that by treating the exfoliated MoS_2_ with sulfur vapor at high temperature (435 °C) under vacuum ambient can cause the sulfur atoms to diffuse into MoS_2_. This has led to an improvement in the S:Mo atomic ratio from 1.89 (as-exfoliated) to ~1.96 after sulfur treatment. With a near ideal stoichiometric S:Mo ratio, the electrical properties such as threshold voltage, current on/off ratio and electron mobility are expected to be improved.

**Figure 5:**
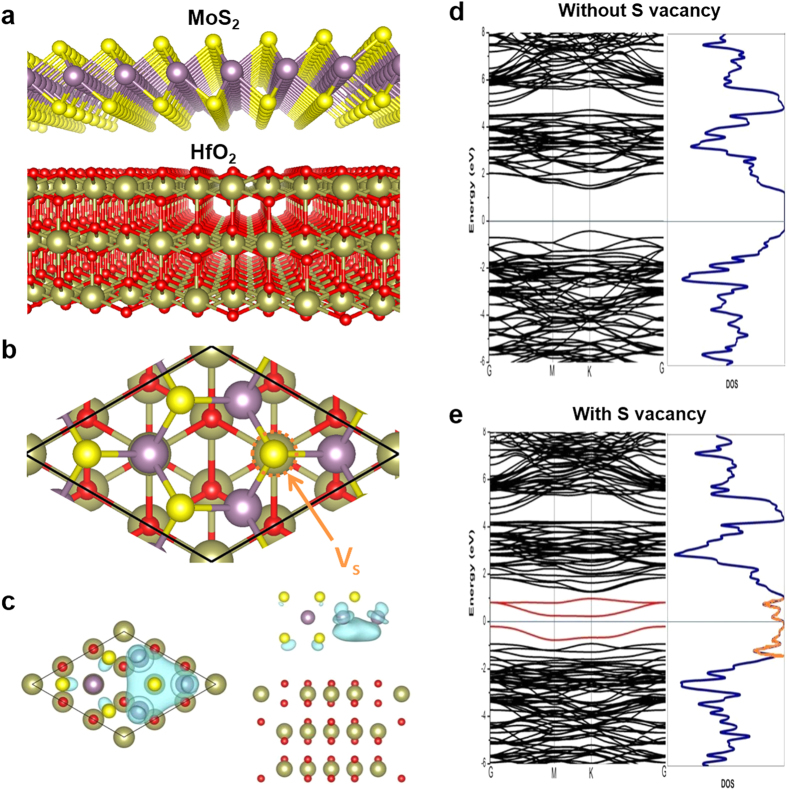
The top and side views of the MoS_2_-HfO_2_ interface structures. (**a**) The perspective side view of the supercell of our model. (**b**) Top view of the unit cell. *V*_*s*_ indicates the location of S vacancy in the interfacial region for the calculation of the case with S vacancy. For MoS_2_-HfO_2_ interface with S vacancy, the marked S atom is removed. S atoms are represented by gold spheres; Mo atoms are represented by purple spheres; O atoms are represented by red spheres; Hf atoms are represented by green spheres. (**c**) Top and side views of real-space charge density of the defect state below the Fermi level at Γ (0, 0, 0). The charge density isosurfaces are taken to be 0.004 e/bohr^3^. (**d**) Band structure and its corresponding density of states (DOS) for the MoS_2_-HfO_2_ interface without S vacancy. Fermi levels are aligned at the origin of the energy scale. (**e**) Band structure and its corresponding DOS of MoS_2_-HfO_2_ interface with S vacancy. Fermi levels are aligned at the origin of the energy scale. The trap states are colored in red in the band structure and DOS, which exist within the MoS_2_ bandgap.

To support our hypothesis, first-principles calculations within the density-functional theory (DFT) framework are performed using the Vienna Ab-initio Simulation Package (VASP)^37^,^38^,^39^. Projector augmented wave (PAW) method^40^,^41^,^42^, and Perdew, Burke, and Ernzerhof (PBE) functional^43^,^44^ with generalized gradient approximation (GGA) are used. We chose cubic HfO_2_ (111) as the substrate due to its simple structure and hexagonal surface unit cell. To eliminate the strain effect, the MoS_2_-HfO_2_ is modeled with a non-pseudomorphic periodic unit cell obtained by superposition of (√3 × √3) R30°-HfO_2_ (111) and (2 × 2)-MoS_2_ monolayer structures. Further details about the calculation are described in the Methods section. Figure 5(a,b) show the perspective side view of the supercell of our model and the top view of unit cell where *V*_*s*_ indicates the location of S vacancy in the interfacial region for the calculation of the case with S vacancy, respectively. S atoms are represented by gold spheres; Mo atoms are represented by purple spheres; O atoms are represented by red spheres; while Hf atoms are represented by green spheres. It is noted that the most stable interfacial configuration is produced when three S atoms of the MoS_2_ monolayer are residing on top of three interfacial Hf atoms. This facilitates the formation of interfacial Hf-S bonds due to the more chemically active Hf atoms that are experimentally verified using XPS measurement, where the Hf *4d*_*3/2*_ and S *2 s* orbitals are found to peak at 224.4 and 226.4 eV, respectively [Fig. 4(b)]. Whereas in the case of MoS_2_-HfO_2_ interface with S vacancy, the marked S atom is removed. The top and side views of the real-space charge density of defect states below the Fermi level at Γ (0, 0, 0) are shown in Fig. 5(c), in which the charge density isosurfaces are taken to be 0.004 e/bohr^3^. Figure 5(d,e) show the band structures and the corresponding density of states (DOS) for the MoS_2_-HfO_2_ interface without and with S vacancy, respectively. The interface states are colored in red in the band structure and DOS, proving that the presence of S vacancy is responsible for the generation of trap states within the MoS_2_ bandgap. Based on first-principles study and considering all values of chemical potentials relevant to the growth of MoS_2_, the S vacancies (*V*_*S*_) are found to be the most abundant defects^45^. Such *V*_*S*_ are also known to be deep acceptor traps that are usually located near midgap. This is consistent with the larger frequency dispersion observed at weak accumulation in our devices, which affirms that a more severe trap states should be present near the mid-gap of monolayer-MoS_2_. Hence when operates in the strong accumulation regime where the Fermi level moves away from the mid-gap traps due to an increased gate voltage, a reduction in the frequency dispersion is observed. These results further strengthen our findings that the frequency dispersion is corroborated to the interface states due to sulfur vacancies that exist at the MoS_2_-HfO_2_ interface.

## Conclusion

The origin of interface states and its impact on the *C-V* frequency dispersion of MOSCAP with monolayer MoS_2_ and HfO_2_ high-*k* dielectric is investigated for the first time. We show experimentally that the presence of sulfur vacancies at the MoS_2_-HfO_2_ interface is responsible for the generation of interface states, as evidenced by the deficit S:Mo ratio through XPS analysis. First-principles calculations further confirm the existence of trap states due to sulfur deficiency that exist within the MoS_2_ bandgap. This accounts for the frequency dispersion measured in the accumulation regime, which demonstrates a dependence on the applied gate voltage. Further improvement in *D*_*it*_ could be achieved by controlling the thermal diffusion of sulfur atoms and its successive passivation with the interface vacancies, leading to a pathway to improve the carrier transport properties of MoS_2_ devices.

## Methods

### Sample Preparation and Device Fabrication

An n-type degenerately doped silicon substrate is used as the back gate electrode. A 5 nm thick HfO_2_ is subsequently deposited by atomic layer deposition (ALD) process. During the growth of the HfO_2_, the Si substrate is also oxidized at the surface to form an interfacial oxide layer. Following that, monolayer MoS_2_ thin film is deposited by magnetron sputtering onto the HfO_2_/Si substrate. The substrate is pre-cleaned using H_2_SO_4_, acetone, and deionized (DI) water in an ultrasonic bath prior to loading into the deposition chamber and pre-heated at 700 °C before the growth^17^. The MoS_2_ films are grown at high temperature (>700 °C) using Mo metal target sputtered in vaporized sulfur ambient. Sulfur is vaporized using heating tape wrapping around the sulfur container before leaking into the chamber. The base pressure of the chamber is 3 × 10^−7^ mbar. The Argon pressure is fixed at 6.0 × 10^−4^ mbar and the sputtering power is as low as 6 W. At this low power, the growth rate is extremely low so as to achieve mono- to few-layers growth. The layer number of the resulting MoS_2_ films can be controlled by tuning the sputtering power and deposition time. Finally, a 100 nm titanium (Ti) is deposited by e-beam evaporation and lifted off to form the metal electrodes.

### Sample Characterization

Raman spectra are obtained on a single-gating micro-Raman spectrometer (Horiba-JY T64000) excited with 532 nm laser. The signal is collected through a 100 × objective lens, dispersed with a grating of 1800 g mm^−1^, and detected by a liquid nitrogen-cooled charge-coupled device. The samples are *in-situ* transferred to an XPS chamber for analysis. XPS measurements are performed in a VG ESCALAB 220i-XL system using a monochromatic Al Kα source. The pass energy of the analyzer is set to 10 eV for high measurement resolution. After that, a layer of photoresist is coated, UV-exposed under a mask and developed out a series of 61 μm × 37 μm rectangular windows.

### Density Functional Theory (DFT) Calculation

DFT is performed using the Vienna Ab-initio Simulation Package (VASP)^37^,^38^,^39^. Projector augmented wave (PAW) method^40^,^41^,^42^, and Perdew, Burke, and Ernzerhof (PBE) functional^43^,^44^ with generalized gradient approximation (GGA) are used. We chose cubic HfO_2_ (111) as the substrate because of its simple structure and hexagonal surface unit cell. In order to eliminate the strain, the MoS_2_-HfO_2_ is modeled with a non-pseudomorphic periodic unit cell obtained by superposition of (√3 × √3) R30°-HfO_2_ (111) and (2 × 2)-MoS_2_ monolayer structures. The substrate contained three O-Hf-O trilayers wherein the top two trilayers are left free to move while the bottom trilayer is frozen at their equilibrium bulk positions to mimic bulk properties, which gave the converged results. The energy cutoff is 450 eV, and the successive slabs are separated by a vacuum layer of about 17 Å. Each atom is relaxed until the residual force acting on each atom is less than 0.02 eV/Å. The (4 × 4 × 1) and (8 × 8 × 1) *k*-point samplings are used for structural relaxations and electronic properties.

## Additional Information

**How to cite this article**: Xia, P. *et al*. Impact and Origin of Interface States in MOS Capacitor with Monolayer MoS_2_ and HfO_2_ High-*k* Dielectric. *Sci. Rep.* **7**, 40669; doi: 10.1038/srep40669 (2017).

**Publisher's note:** Springer Nature remains neutral with regard to jurisdictional claims in published maps and institutional affiliations.
